# Evaluation of *Cyanea capillata* Sting Management Protocols Using Ex Vivo and In Vitro Envenomation Models

**DOI:** 10.3390/toxins9070215

**Published:** 2017-07-07

**Authors:** Thomas K. Doyle, Jasmine L. Headlam, Christie L. Wilcox, Eoin MacLoughlin, Angel A. Yanagihara

**Affiliations:** 1 Discipline of Zoology, School of Natural Sciences, Ryan Institute, National University of Ireland Galway, Galway H91 W5P7, Ireland; J.HEADLAM1@nuigalway.ie (J.L.H.); eoin.macloughlin@nuigalway.ie (E.M.); 2 Department of Tropical Medicine, Medical Microbiology and Pharmacology, John A. Burns School of Medicine, University of Hawaii at Mānoa, Honolulu, HI 96813, USA; wilcoxcl@hawaii.edu; 3 Békésy Laboratory of Neurobiology, Pacific Biosciences Research Center, School of Ocean and Earth Science and Technology, University of Hawaii at Mānoa, Honolulu, HI 96822, USA

**Keywords:** Scyphozoa, cnidarian envenomations, first aid, hair jelly

## Abstract

Lion’s mane jellyfish (*Cyanea capillata*) stings cause severe pain and can lead to dangerous systemic effects, including Irukandji-like syndrome. As is the case for most cnidarian stings, recommended medical protocols in response to such stings lack rigorous scientific support. In this study, we sought to evaluate potential first aid care protocols using previously described envenomation models that allow for direct measurements of venom activity. We found that seawater rinsing, the most commonly recommended method of tentacle removal for this species, induced significant increases in venom delivery, while rinsing with vinegar or Sting No More^®^ Spray did not. Post-sting temperature treatments affected sting severity, with 40 min of hot-pack treatment reducing lysis of sheep’s blood (in agar plates), a direct representation of venom load, by over 90%. Ice pack treatment had no effect on sting severity. These results indicate that sting management protocols for *Cyanea* need to be revised immediately to discontinue rinsing with seawater and include the use of heat treatment.

## 1. Introduction

Jellyfish of the genus *Cyanea* are widely distributed in temperate, boreal and polar waters of the Pacific and Atlantic Oceans [1,2,3,4,5]. While all *Cyanea* species are known to be venomous, the lion’s mane jellyfish (*Cyanea capillata*) has been medically problematic for at least 100 years [6]. It is a large jellyfish (up to 1 m bell dimeter) with eight groups of ~100 tentacles each located on the subumbrellar side of the jellyfish [7]. In Irish and UK waters, lion’s mane jellyfish can be encountered from June until late September [4]. It is one of the least abundant jellyfish in Irish and UK waters, typically occurring as single individuals rather than in blooms or aggregations [4,8]. Despite being one of the least abundant jellyfish, relatively high densities of lion’s mane jellyfish have been recorded close to high population centers (e.g., Dublin Bay), making stings a frequent problem. Furthermore, open-ocean swimming is very popular in the UK and Ireland, and many swimming clubs and events are held in areas where lion’s mane jellyfish are known to be abundant, and therefore stings are a recurrent concern. Over the past 10 years, there have been several beach closures due to the lion’s mane jellyfish and at other times signs have been put in place warning bathers that the water is not safe to swim because of the lion’s mane jellyfish. Indeed, during a previous study in this area, 51% of bathers (*n* = 77) said that they had been badly stung by a jellyfish, and three said they required treatment at a hospital (T.K.D., unpublished data). While no central database exists in Ireland documenting the numbers of sting incidents requiring medical attention, it is likely between 10 and 100 persons per year (T.K.D., pers. obs.).

*Cyanea* stings, though not generally considered fatal, can cause severe local reactions, including extreme pain and edema, as well as systemic symptoms and clinical signs [9,10,11,12,13,14]. Envenomations involving large specimens can be particularly dangerous, as the thousands of almost invisibly thin tentacles can each extend to several meters long. Initial dermal contact may result in itching or localized pain that may radiate to other areas of the body, potentially progressing to severe pain within 20 min or more. Weakness, vertigo, nausea, headache, muscle cramps, lacrimation and perspiration may also occur. In very severe stings, there may be difficulty breathing and pain on respiration, tachycardia, muscle spasms and stiffness of back and joints. The skin may become red with urticarial weals, local edema, blisters and weeping of the skin, which may progress to ulceration and secondary infection. In some cases, stings can result in Irukandji-like syndrome (i.e., symptoms include back “pain, nausea, abdominal cramps, sweating, hypertension, tachycardia and a feeling of impending doom” and usually develop 20–60 min after a sting [15]).

Currently, sting management protocols suffer from a lack of rigorous evidence-based support. For example, a recent literature review [16] found very few studies evaluating recommended sting protocols for species found in German waters (including *C. capillata*), and those that were identified were classified level 4 or less on the evidence classification scale, as described by the Oxford Centre for Evidence-Based Medicine (CEBM) [17]. Previous reviews have similarly found scant evidence supporting first aid methods for *C. capillata* stings [18,19]. For *C. capillata* specifically, only two studies have been conducted which evaluate the efficacy of potential first aids [20,21]. The only study examining potential removal methods was conducted more than 30 years ago, relied solely on in vitro examination of nematocyst discharge in response to potential rinse solutions, and did not include quantitative results, raw data images, or statistical comparisons between treatments [21]. Recent work has demonstrated that nematocyst discharge in vitro has limited (if any) correlation to sting severity as measured by direct functional assays [22] or human clinical trials [23]. Similarly, the only study supporting the use of cold packs for pain relief was uncontrolled and contained no statistical analysis [20].

Despite the dearth of studies evaluating the effects of potential interventions, most authorities currently recommend that *C. capillata* tentacles be removed by rinsing with seawater/saline [13,18,24,25,26] (references stating that vinegar rinsing is specifically contraindicated [9,18,24]), and that the sting site should be treated with either hot water immersion/heat [24,25], cold packs/ice [18,26], or a baking soda slurry [9,25,27,28]. In the clinical literature, medical databases and lay-level advice articles, species-specific recommendations are often not given; instead, general recommendations are made for all ‘jellyfish’ (sometimes limited by geographic area) [26,29,30,31], all scyphozoan species (sometimes lumped as ‘sea nettles’) [27,32], or all non-tropical and/or non-cubozoan species [33,34,35]. For these reasons evidence based research utilizing direct activity assays are urgently needed to systematically evaluate medically relevant species.

The purpose of this study was to re-evaluate common first aid recommendations for *C. capillata* stings using a combination of in vitro discharge tests, as well as envenomation models which evaluate functional venom activity [36]. Potential rinse solutions (seawater, urine, vinegar, and Sting No More^®^ Spray), as well as temperature treatments (hot packs versus ice packs) were evaluated.

## 2. Results

### 2.1. Testing of Potential Rinse Solutions Using the Tentacle Solution Assay

**Figure 1:**
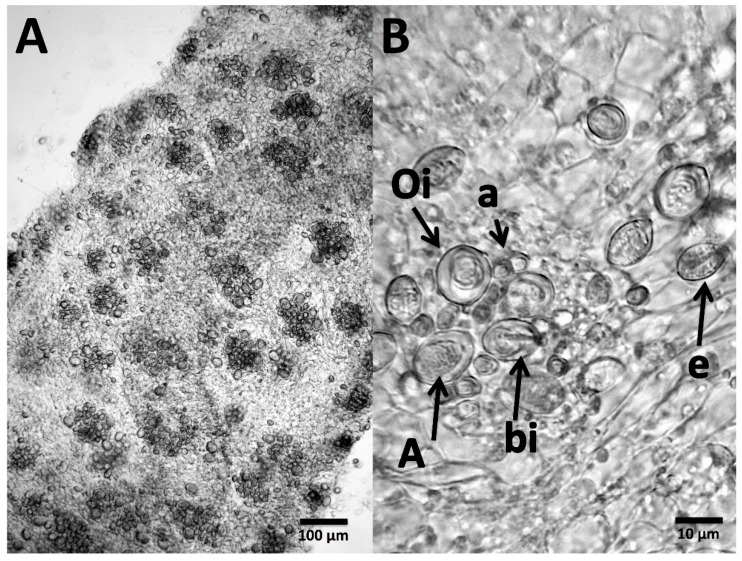
*Cyanea* tentacles viewed microscopically. (**A**) Tentacle viewed at 10× magnification; discrete ‘batteries’ of cnidae can be seen, each containing a mix of different cnidae types. (**B**) 40× magnification of a typical tentacle battery showing the complex and difficult to identify cnidome; cnidae identified according to [37]. a, a-isorhizas; A, A-isorhiza; e, euryteles; Oi, O-isorhizas; bi, birhopaloids.

To compare with previous investigations and studies conducted on other species, we examined the in vitro effects of potential rinse solutions using the Tentacle Solution Assay (TSA) [36]. However, precise quantification of nematocyst discharge in response to test solutions proved difficult given the complexity of the *Cyanea* cnidome, which consists of euryteles, birhopaloids and three different isorhizas—a-isorhizas, A-isorhizas, and O-isorhizas—each with multiple size classes [37] (Figure 1). It is essential to distinguish these types when evaluating discharge because studies have demonstrated that different nematocysts can vary not only in their morphology and penetrant abilities but also in their toxic effects [38,39,40]. However, precise quantification of percent discharge for each nematocyst type and size class was not possible with the microscope and camera available for this study; best efforts were made to identify cnidae types based on [37]. Further, it is also likely that some of the cnidae visualized in this study are immature, particularly smaller cnidae [37]; it was not possible to distinguish immature versus mature cnidae in this study.

**Figure 2:**
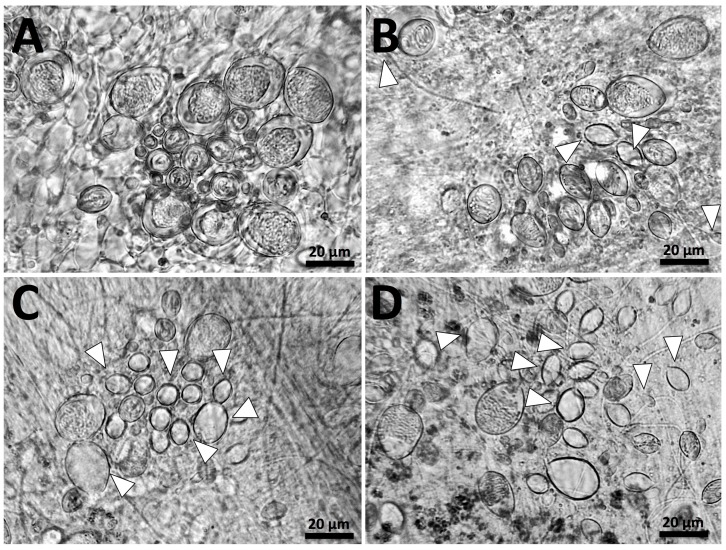
*Cyanea* cnidae discharge in response to (**A**) seawater; (**B**) vinegar; (**C**) urine; (**D**) isopropanol. No discharge can be seen in (**A**), while (**B**–**D**) all contain discharged cnidae (examples indicated with white arrows). However, the discharge in (**B**) is only partial (<20%) and limited to putative a-isorhizas and euryteles, while (**C**,**D**) show moderate discharge (~50%) of all cnidae types.

Seawater is an inert solution, and did not elicit discharge (Figure 2A). When tentacles were treated with vinegar (Figure 2B), some discharge of cnidae did occur, but discharge was not equally distributed between cnidae types. Cnidae identified as a-isorhizas and euryteles (or possibly immature cnidae, based on their size) discharged to some extent (<20% discharge; white arrows), but A-isorhizas, O-isorhizas and birhopaloids largely did not (estimated <5% discharge of each). In contrast, the application of urine (Figure 2C) and isopropanol (Figure 2D) induced ~50% discharge in all cnidae types. Pressure also led to ~50% discharge of all cnidae types (data not shown). Therefore, while these results are qualitative rather than quantitative, there were clear differences between the different treatments with no response from sea water, mostly <5% firing of cnidae for vinegar (except for a-isorhizas and euryteles which had a <20% firing rate) and then ~50% firing rate for all other treatments: urine, isopropanol and pressure.

**Figure 3:**
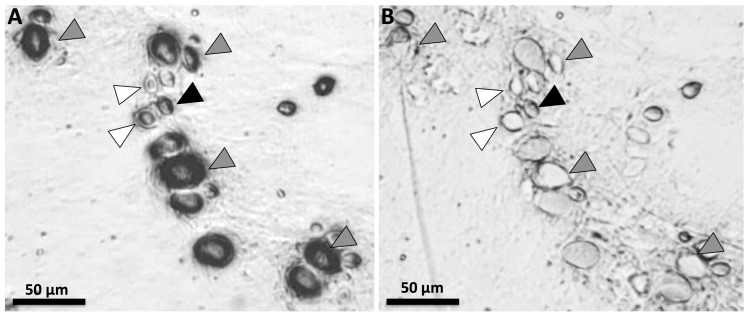
Nematocysts remaining on 5% gelatin after a 5 min *C. capillata* sting before (**A**) and after (**B**) vinegar application. White arrows indicate discharged, penetrant nematocyst and black arrows indicate non-discharged nematocysts that did not discharge at any point during the experiment. Grey arrows point to nematocysts that discharged in response to vinegar application; however, in all cases, tubules could be seen everting upwards into the vinegar droplet and not penetrating the gelatin below, suggesting that no additional venom delivery into the gelatin in response to vinegar application despite the solution causing nematocyst discharge.

Since previous studies have found discrepancies between discharge seen in vitro and venom activity in functional assays [22], the effects of vinegar on cnidae discharge were further examined in a stinging model. Tentacles were placed upon 5% gelatin and remained in place for 5 min. Tentacles were then pulled off with tweezers, and adherent cnidae were examined microscopically to discern cnidae type, relative abundances and proportional type-specific discharge. Among the adherent cnidae, smaller size classes were more likely to be discharged (Figure 3A, white arrows). While recording video (see Video S1), vinegar was added directly to the adherent cnidae to evaluate whether vinegar application induced any specific morphological response. This was done to address the question as to whether vinegar rinsing could result in functional discharge (i.e., envenomation) if used as a post-sting rinse (before vinegar, Figure 3A; after vinegar, Figure 3B). While many cnidae did not react to vinegar (e.g., Figure 3, black arrow), specific cnidae types evidenced morphological responses (Figure 3, grey arrows). It should be noted that while intact cnidae capsules respond to various stimuli, the response can include non-everting rupture, partial eversion discharge, and fully everting discharge in which the tubule productively impales the substrate (prey tissue) as well as eversions in which the tubule non-productively discharges into the surrounding seawater. For this reason it is noteworthy that among cnidae that discharged in response to vinegar application, all of the tubules appeared to evert non-productively upwards into the vinegar droplet and did not impale the gelatin (Figure 3B, grey arrows; Video S1). Thus, no envenomating discharge of cnidae was observed in response to vinegar application, suggesting that the subpopulation of cnidae that are triggered to discharge in response to the application of vinegar are either not penetrant venom-laden nematocysts, or that if they are nematocysts, this type of capsule rupture does not result in functional venom delivery. Instead, vinegar application may essentially inactivate this subpopulation of cnidae by inducing aberrant capsule rupture rather than authentic trauma inducing discharge.

### 2.2. Testing of First Aid Measures Using Functional Models

In addition to solution-based assays, functional venom activity assays were conducted using live, spontaneously stinging tentacles [36]. We sought to evaluate whether the rinse solutions tested in TSAs as well as post sting topical hot-, ambient- or cold-pack exposure (for which there is no method for testing using TSAs) led to increases or decreases in hemolytic zone formation, a function metric of venom activity or “venom load”.

**Figure 4:**
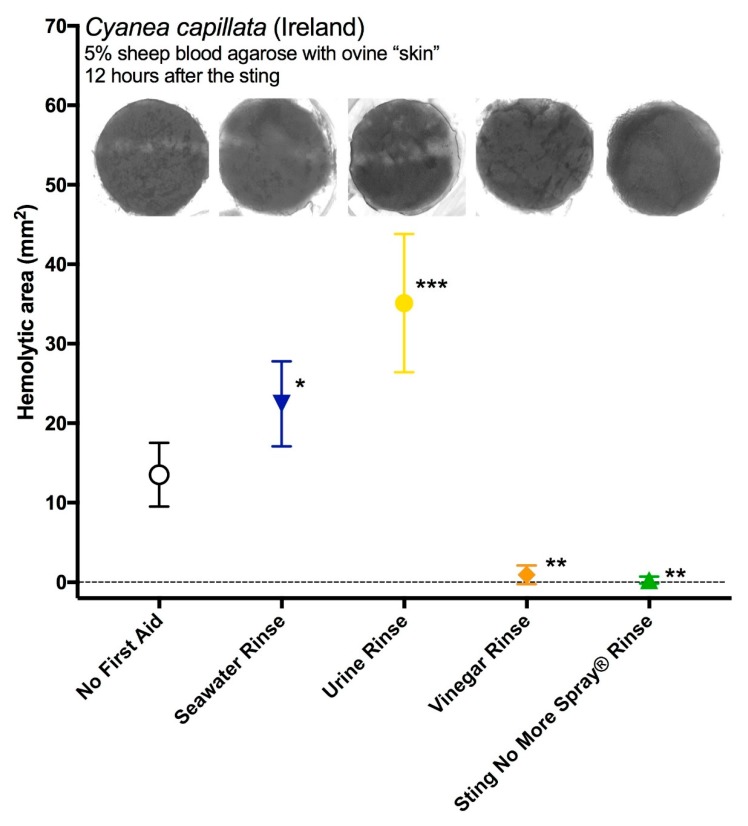
Size of venom-induced hemolytic zone after 12 h using the TSBAA model (sheep’s blood agarose) when *C. capillata* tentacles were removed by rinsing with seawater (dark blue), urine (yellow), vinegar (orange) or Sting No More^®^ Spray (green); *N* = 3 for each, points represent mean ± standard error. The addition of seawater or urine significantly increased the size of the hemolytic area (*p* = 0.0350 and 0.0001, respectively in Fisher’s LSD post-hoc analyses), while the use of vinegar or Sting No More^®^ Spray reduced hemolysis (*p* = 0.0061 and 0.0045, respectively); asterisks denote level of significance from no first aid: one asterisk for *p* < 0.05, two for *p* < 0.01 and three for *p* < 0.001.

Different rinse solutions significantly affected the size of the hemolytic zone after 12 h (one-way ANOVA, *p* < 0.0001; Figure 4). When compared with simply pulling off tentacles, the application of urine or seawater significantly increased hemolysis (*p* = 0.0350 and 0.0001, respectively in Fisher’s LSD post-hoc analyses), while the use of vinegar or Sting No More^®^ Spray reduced hemolysis (*p* = 0.0061 and 0.0045, respectively). These results conflict our TSA results, which found that seawater was “inert” (did not induce discharge) while vinegar did induce some cnidae discharge (Figure 2B, Figure 3), suggesting that the in vitro studies do not accurately predict actual venom delivery during stings.

**Figure 5:**
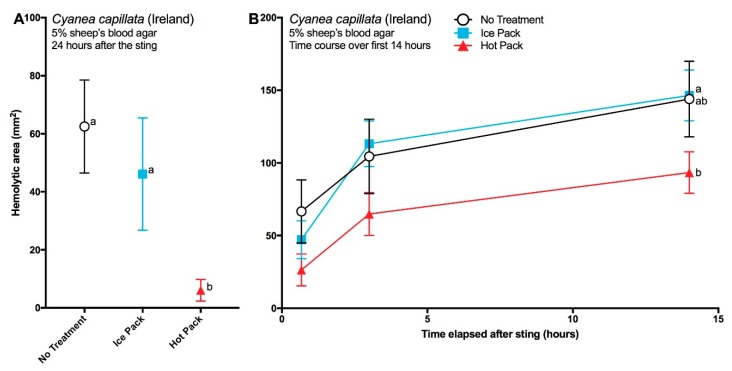
Size of venom-induced hemolytic zone from two experiments using the Tentacle Skin Blood Agarose Assay (TSBAA) model (sheep’s blood agar) when stings were treated with hot packs (red triangles), ice packs (blue squares), or kept at room temperature (circles); *N* = 6 for all, points represent mean ± standard error. (**A**) Experiment 1 was carried out on the 27th July 2016 and the hemolytic zone was reported after 24 h and in (**B**) Experiment 2 was carried out on 4th August 2016 and the hemolytic zone was measured at three time periods (40 min, 3 h and 14 h). In experiment 1, there were significant differences between the treatments after 24 h (one-way ANOVA, *p* < 0.0001), with hot packs significantly reducing the hemolytic area size when compared with either ice packs (*p* < 0.0001, Fisher’s LSD) or no temperature treatment (*p* = 0.0004). In experiment 2, a significant difference was detected at the 14 h time period between hot and cold treatments (*p* = 0.0483, Two-Way ANOVA with post-hoc Fisher's LSD) using a two-way ANOVA.

The application of post-sting temperature treatments for 40 min also significantly affected sting severity (Figure 5). Over time, greater differences in hemolysis were seen between hot packs and ice packs (Figure 5B), resulting in significantly less hemolysis from stings treated with hot packs after 24 h (Figure 5A). All three treatments in hot/cold experiment 2 (Figure 5B) showed much greater hemolysis than the first experiment (Figure 5A). No differences were detected between stings that did not receive a temperature treatment and those that received ice.

## 3. Discussion

The current recommendations for treating *Cyanea* stings vary greatly depending on the source. Most recommend rinsing the sting site with seawater or saline [13,18,24,25,26] followed by hot water immersion/heat [24,25], cold packs/ice [18,26], or a baking soda slurry [9,25,27,28]. We did not evaluate the last option in this study. However, we found that seawater rinsing increased functional venom delivery or “venom load” and that there was no benefit from the use of ice. Instead, our results support the use of vinegar to rinse away adherent tentacles and treatment with at least 40 min of 45 °C heat to limit injected venom activity.

It may seem surprising that rinsing with vinegar led to significant decreases in venom activity (Figure 4) given that it induces some cnidae discharge in vitro (Figure 2 and Figure 3). In our study, we noted that vinegar does not equally induce discharge in all nematocyst types, and did not elicit as much discharge as other solutions (urine, isopropanol). It is not known how each nematocyst type contributes to toxicity, but previous studies have suggested that the largest A-isorhizas and O-isorhizas disproportionately contribute to hemolysis [40], and these cnidae types were not triggered by vinegar application. Thus, it may be that vinegar does not induce discharge of the most toxic types of nematocysts, and that any discharge that occurs during the rinsing process does not contribute measurably to hemolytic activity, the functional metric of our venom activity assays. Or, as seen when vinegar was applied to adherent cnidae in our simple gelatin sting model (Figure 3), it is possible that vinegar application induces agonal, biologically inactive discharge (as suggested by Auerbach [41]), rendering cnidae incapable of functionally delivering venom. Upon tentacle contact with skin, a certain percentage of cnidae immediately discharge. However, a great number of cnidae were found to have been transferred to the skin intact. Because sea water does not irreversibly inhibit cnidae discharge, cnidae left at the contact site retain the capacity to discharge and thus as the tentacle rolls along the skin during the sea water rinse, additional undischarged cnidae are transferred beyond the original “sting” site. Finally, the data in this study demonstrate that these residual undischarged cnidae discharge spontaneously over the time course examined, to result in a greater area of a hemolytic zone as shown is Figure 4. Urine, a commonly believed folk remedy for jellyfish stings, was even worse than seawater (Figure 4), which aligns with out TSA data showing it elicits ~50% discharge of all cnidae types. These results stress the importance of evaluating first aid protocols using functional activity assays rather than solution-only tests, as they add to a growing number of studies that have shown in vitro examinations are not necessarily predictive of clinically relevant effects [22,23,42].

Overall the hot pack treatment reduced sting severity despite the observed variability in venom potency between the two experiments (Figure 5A,B). This may reflect individual animal variability in potency or differences in the duration of time between animal capture at sea and the removal of tentacles for the experiments. Nonetheless, the observed reduction of venom activity by heat (Figure 5) is in concurrence with similar studies in hydrozoans [42] and cubozoans [22,36] and the body of clinical literature that demonstrates improved clinical outcomes with heat application as well as the low level of heat thermotolerance of cnidarian venoms (for a review see [43]). Some have suggested that improved outcomes from heat application (in particular the reduction of pain seen in clinical studies [44,45,46]) are not the result of reduction in venom activity, but instead, reflect modulation of neurological pain processing [47]. This is directly disputed by the results of our envenomation modeling, where we demonstrate a direct dampening effect of heat application on venom activity in a model system that lacks any neurons or neural pathways. And as similar results have been achieved across three separate cnidarian classes, these data suggest that cnidarian venoms in general are heat-sensitive and that the sustained application of heat (at least 40 min), in the form of 45 °C hot packs or hot water immersion, is an effective first aid for reducing the damage caused by injected venom. Indeed recent research has shown that a crude venom extract from *Pelagia noctiluca* (another scyphozoan jellyfish) also exhibits a loss of potency at temperatures higher than 40 °C [48,49]). Similarly, biochemical studies have shown marked 45 °C heat related loss of activity in vitro [50,51,52,53,54,55,56,57,58,59,60,61,62,63,64] which may reflect thermal unfolding or aggregation. Additional research is needed to determine exactly why heat has this direct, negative effect on venom activity; there may be less evolutionary pressure for heat tolerance among cnidarian venom proteins than mammalian proteins. Taken together, the observation that venom protein activities are significantly inhibited at tolerably hot temperatures far below those required to induce mammalian protein biophysical denaturation, (i.e., measurable loss of tertiary or secondary structure) provides the basis for safe first aid reduction of the activity of lytic components.

Because of the great diversity of stinging jellyfish (cubozoans, hydrozoans and scyphomedusae), it has been previously stated that different jellyfish may require different treatments [9,65]. Building on previous work on box jellyfish [22,43] and the Portuguese man o’ war [42], this study now shows that jellyfish from three different classes of Cnidaria (Cubozoa, Hydrozoa and Scyphozoa) respond in the same way to the application of vinegar (despite slight differences in response to vinegar in vitro) and heat. This will therefore, simplify the development of a first aid protocol for jellyfish stings even in countries that have several very different venomous jellyfish species.

## 4. Conclusions

As *C. capillata* envenomations represent a significant medical burden worldwide, it is important that evidence-based medical treatments be employed when generating first aid management protocols. We found that despite inducing some detectable cnidae discharge in vitro, vinegar was the most effective non-commercial rinse solution for safely removing adherent tentacles and nematocysts. The commercial product Sting No More^®^ Spray was equally effective, while the use of seawater and urine exacerbated stings. We also found the application of a 40-min, 45 °C hot pack reduced the activity of successfully injected venom, and thus worked well as a treatment. Because our model does not include metrics for pain or neurological processes, we are able to affirm that heat has a direct effect on venom proteins rather than an indirect, modulating effect on pain sensory systems. Heat application reduced the activity of injected venom, while the application of ice had no significant effect. Thus, we conclude that the best first aid for *C. capillata* stings is a two-step protocol of (1) rinsing with vinegar or Sting No More^®^ Spray and (2) 40 min or longer treatment with hot packs or hot water immersion (45 °C/113 °F).

## 5. Materials and Methods

The chemicals for the solutions used in all assays are as follows: seawater (locally collected), freshwater (tap water), distilled white vinegar (Tesco, produced in the UK for Tesco Stores Ltd., Chestnut, UK for experiments conducted in Ireland; Market Pantry, Target Corporation, Minneapolis, MN, USA for those conducted in the USA), isopropanol (91%, Up & Up, Target Corporation, Minneapolis, MN, USA), Sting No More^®^ Spray (contents include vinegar, copper gluconate, urea, and magnesium sulfate; Alatalab Solutions™ LLC, Honolulu, HI, USA), and gelatin (Knox^®^ Gelatine, Kraft Food Groups, Inc., Northfield, IL, USA). Urine was freshly collected from a willing volunteer (pH 5.25).

### 5.1. Animal Collection

For in vitro examination, tentacles were collected just prior to experiments from live *C. capillata* harvested from Puget Sound and kept in aquaria at the Point Defiance Zoo and Aquarium in Tacoma, WA. Animals had spent approximately one year in captivity prior to experiments. For functional assays involving blood cells, live *C. capillata* were collected from Dublin Bay (between the Forty Foot bathing area and Dalkey Island, 53.288402° N, −6.103343° W). Animals were placed in individual 50 L containers full to the brim with seawater to prevent damage to the animals from sloshing during transport. Tentacles were harvested just prior to their use in experiments; all experiments were conducted within 72 h of collection.

### 5.2. Tentacle Solution Assay (TSA) and In Vitro Tests

To test for the induction of discharge, freshly cut tentacles (length 1–2 cm) from a captive specimen of *C. capillata* housed at the Point Defiance Zoo and Aquarium were placed on clean, dry microscope slides and examined quickly for discharge; any lengths with notable discharge were discarded [36]. 60 μL of the test solution was then added to the tentacle. Test solutions were (a) sea water, (b) vinegar, (c) urine and (d) isopropanol (Sting No More^®^ Spray was not used during the test as it was not available). After one minute of incubation, a cover slip was gently placed over the tentacle. Preliminary tests with seawater confirmed that coverslip addition did not induce significant discharge. All photos were taken of the tentacles ten minutes after the various treatments through the coverslip using a compound microscope at 10× and 40× magnification (microscope: OMAX M837ZL Compound Microscope, OMAX, Bucheon, South Korea; camera: OMAX A35140U; photo software: ToupLIte vers. 1.0., ToupTek, Zhejiang, China).

To further evaluate discharge in response to vinegar, tentacles were allowed to sting a 2 mm thick slab of 5% gelatin in seawater for five minutes. After tentacles were pulled off using tweezers, adherent cnidae were examined microscopically at 10× magnification. A video was taken as vinegar was applied to an area with both discharged and undischarged cnidae, and one minute of footage was recorded. Video footage was examined to determine whether vinegar application induced any nematocysts in the field of vision to discharge and, if so, whether the everting tubules penetrated the gelatin substrate (microscope: AmScope M158C-E Compound Monocular Microscope, AmScope, Irvine, CA, USA; camera: AmScope MD35; photo software: Proscope HR version 1.2.1., Bodelin, Wilsonville, OR, USA).

### 5.3. Evaluating First Aid Measures with Functional Assays (Tentacle Skin Blood Agarose Assay, or TSBAA)

The effect of solutions and temperature treatments on sting severity was evaluated using a “skin” covered adaptation of the Tentacle Blood Agarose Assay (TBAA) ex vivo envenomation model outlined in [36]. Briefly, premade sheep’s blood agar plates (Remel™, Lenexa, KS, USA) were used for the Tentacle Skin Blood Agarose Assay (TSBAA).

For rinse experiments, 15 blood agarose rounds were extracted from the blood agar plates (20 mm diameter). These rounds were placed on top of a layer of cling film laid over the open end of a glass jar (diameter approx. 45 mm). Sections of the prepared ovine intestine were laid over the agar rounds. The lids of the glass jars had a hole approx. 25 mm cut into them and these lids were placed on top of glass jar, cling film, agar and skin. Fresh tentacles were allowed to sting for 3 min before the test solutions were applied directly onto the tentacles using a spray bottle. Test solutions were: (a) control (no solution), (b) sea water, (c) vinegar, (d) urine and (e) Sting No More^®^ Spray. Isopropanol was not used as there were only 15 agarose rounds due a shortage of ovine intestines during the experiment, and based on previous research the authors wanted to test the effectiveness of Sting No More Spray as a potential rinse solution. The tentacles remained for another 2 min before the skins and tentacles were removed. Photos were taken after 40 min and 12 h of incubation at room temperature.

To evaluate temperature treatments, 9 blood agar plates were acclimated to room temperature. Two tentacles were added to each blood agar plate and allowed to sting for three minutes before they were pulled off using tweezers. 3 plates each received (a) no temperature treatment (control); (b) an ice pack for 40 min; and (c) a 45 °C hot pack for 40 min (thus a total of *N* = 6 stings for each condition). Plates were incubated at room temperature. In the first experiment, photos were taken at 40 min and 24 h. The experiment was then repeated; however, for the second experiment, photos were taken at 40 min, 3 h and 14 h.

### 5.4. Statistical Analyses

For blood agar experiments, the area of the zone of hemolysis was calculated using ImageJ (the United States National Institutes of Health, Bethesda, MD, USA). Briefly, the image scale was set using the known widths and subsections (50 mm × 15 mm or 15 mm × 7.5 mm) were taken from each replicate for analysis to remove edge effects. Controls were used to set the color threshold for no hemolysis. The total area of the hemolytic zone was taken directly from the “analyze particles” function. Hemolytic zone was evaluated as the area exhibiting >80% hemolysis. Shapiro-Wilk normality tests were conducted on the single timepoint data sets; since data were normally distributed, one-way ANOVAs were used. Two-way ANOVAs were used for multiple timepoint data sets. All statistical analyses and post-hoc multiple comparisons were conducted in GraphPad Prism version 6.0 (GraphPad Software, Inc., La Jolla, CA, USA).

## Supplementary Materials

Zenodo DOI:10.5281/zenodo.832574 https://zenodo.org/record/832574, Video S1: Vinegar Application to Gelatin-Adherent Cnidae.

## Abbreviations

MDPI	Multidisciplinary Digital Publishing Institute
TSA	Tentacle Solution Assay
CEBM	Centre for Evidence-Based Medicine
TBAA	Tentacle Blood Agarose Assay
TSBAA	Tentacle Skin Blood Agarose Assay
